# Scratch that itch: revisiting links between self-directed behaviour and parasitological, social and environmental factors in a free-ranging primate

**DOI:** 10.1098/rsos.160571

**Published:** 2016-11-02

**Authors:** Julie Duboscq, Valéria Romano, Cédric Sueur, Andrew J. J. MacIntosh

**Affiliations:** 1 Kyoto University Wildlife Research Center, Kyoto, Japan; 2 Kyoto University Primate Research Institute, Inuyama, Japan; 3 Département Ecologie, Physiologie et Ethologie, Centre National de la Recherche Scientifique, Strasbourg, France; 4 Institut Pluridisciplinaire Hubert Curien, Université de Strasbourg, Strasbourg, France

**Keywords:** scratching, self-grooming, lice load, social behaviour, environment, Japanese macaque

## Abstract

Different hypotheses explain variation in the occurrence of self-directed behaviour such as scratching and self-grooming: a parasite hypothesis linked with ectoparasite load, an environmental hypothesis linked with seasonal conditions and a social hypothesis linked with social factors. These hypotheses are not mutually exclusive but are often considered separately. Here, we revisited these hypotheses together in female Japanese macaques (*Macaca fuscata fuscata*) of Kōjima islet, Japan. We input occurrences of scratching and self-grooming during focal observations in models combining parasitological (lice load), social (dominance rank, social grooming, aggression received and proximity), and environmental (rainfall, temperature and season) variables. Using an information-theory approach, we simultaneously compared the explanatory value of models against each other using variation in Akaike's information criterion and Akaike's weights. We found that evidence for models with lice load, with or without environmental–social parameters, was stronger than that for other models. In these models, scratching was positively associated with lice load and social grooming whereas self-grooming was negatively associated with lice load and positively associated with social grooming, dominance rank and number of female neighbours. This study indicates that the study animals scratch primarily because of an immune/stimulus itch, possibly triggered by ectoparasite bites/movements. It also confirms that self-grooming could act as a displacement activity in the case of social uncertainty. We advocate that biological hypotheses be more broadly considered even when investigating social processes, as one does not exclude the other.

## 1. Introduction

Self-grooming, scratching, rubbing or wallowing are forms of body care behaviours in which many animals engage frequently. In the literature, the frequency of these self-directed behaviours (SDB) has been linked to various factors, such as ectoparasite loads [1–5], environmental conditions [6–9] and social situations [10–16].

Ectoparasites are common parasites of many animals. Even on a small scale, infestation by ectoparasites such as lice, ticks and fleas can cause dermatitis, pruritis (itching), skin sensitization and other allergic reactions. Bites, stings, movements, released chemicals or body parts (e.g. urticating hair) of ectoparasites usually trigger an ‘immune’ or ‘stimulus’ itch [1,17,18]. Although ectoparasites can be susceptible to the immunological system of the host [19], anti-ectoparasite strategies nonetheless tend to involve non-immunological defences such as body care [2–5]. Experimentally preventing animals from grooming themselves or from being groomed generally leads to sharp increases in ectoparasite infestation [2–5], whereas decreasing ectoparasite loads (e.g. by administering anti-parasite drugs) drives reductions in social and self-grooming and scratching [20]. The prophylaxis or parasitic hypothesis thus predicts that the frequency of self-directed behaviour is directly linked to ectoparasite loads [2–5].

A major alternative hypothesis, at least in human and non-human primates, is the anxiety or social hypothesis, which instead links the frequency of SDB to indicators of emotional states and postulates that SDB function to mediate anxiety. SDB in long-tailed macaques are increased by administration of anxiogenic drugs and decreased by that of anxiolytic drugs [21]. Rates often increase in situations of social uncertainty linked to social (particularly aggressive) interactions, uncontrollable/unpredictable proximity of group members, or relative dominance rank [10–16,22,23]. High scratching frequency has also been linked to high degrees of restlessness [24,25], a symptom of generalized anxiety disorder in humans (e.g. [26]).

Increased frequency of body care has also been related to high ambient temperatures and humidity or rainfall [6–9]. Underlying mechanisms behind this environmental hypothesis are often linked to ectoparasite load because the life cycle of many ectoparasites is also influenced by environmental seasonality and their abundance thus fluctuates seasonally [8,27,28]. As the mammalian pelage constitutes the habitat of their ectoparasites, variation in its quality should greatly influence ectoparasite fitness and population dynamics [1], thereby creating the potential for pelage-associated variation in SDB frequency due to habitat-associated effects on ectoparasite loads. At the same time, however, variation in hair length and density also most probably influences the amount of time that animals devote to pelage care for thermoregulation [29], making it difficult to determine whether SDB frequency relates to ectoparasites or some other unrelated ecophysiological factor. Other factors such as sweating or pilo-erection could also play a role but have rarely been investigated [9,29].

To our knowledge, hypotheses relating to whether frequencies of SDB are better explained by one or a combination of the parasite, social and environmental hypotheses have previously not been considered together. To deepen our understanding of the underlying mechanisms and supposed functions of SDB, we simultaneously tested hypotheses explaining rates of such behaviours in female Japanese macaques (*Macaca fuscata fuscata)* of Kōjima islet, Japan. In particular, the possibility that variation in ectoparasite loads with seasonal factors may be an important predictor of SDB in primates has been largely dismissed, and in general, the role of ectoparasite loads has received little consideration in the primate SDB literature. Ectoparasites known to infest Japanese macaques include two species of lice (*Pedicinis obtusus* and *P. eurygaster)* [30] and one species of tick (*Haemaphysalis longicornis*) [31]*.* Lice and louse eggs are commonly observed on Japanese macaques during physical examination [30,31]. A previous study has shown that 98.9% of what individual macaques pick out of the hair while grooming themselves or others, using a very conspicuous sequence of behaviour, is louse eggs [30]. Ticks on the other hand are rarely found on Japanese macaques [30,31], and the gestural sequence of removing them when found differs from that of picking lice [30]. Fleas comprise another group of common ectoparasites, but have not been reported to infest Japanese macaques [30,31].

Thus, most of what is known about ectoparasites of Japanese macaques involves lice. Body parts estimated to have many louse eggs are generally inaccessible and cannot be self-groomed, and are thus socially groomed longer than other body parts [31,32]. The number of louse eggs present on a macaque was also estimated to correspond to approximately double that of the nymph and adult louse population [31], which are the stages that feed obligately on blood. Finally, lice loads estimated from louse egg-picking gestures during grooming were recently shown to vary seasonally in Japanese macaques, although the socio-ecological factors underlying such variation remain to be determined conclusively [33]. Japanese macaques live in a seasonal environment with substantial variation in temperature and rainfall throughout the year [34] and they moult seasonally, with hair being shorter and sparser in summer and longer and denser in winter [35], which could contribute to this observed variation.

In this study, we used an information-theory framework to examine simultaneously and objectively seven mutually non-exclusive hypotheses (formulated as statistical models) related to the occurrence of SDB. Depending on the level of support for each hypothesis among a candidate set, we interpreted the effect of the examined factors on SDB. Specifically, we tested a *parasite hypothesis* that SDB are best explained by lice load alone. Because louse egg removal by self-grooming should be prophylactic, i.e. it removes future blood-feeding, potentially infectious stages from the population, the occurrence of self-grooming and lice load should be negatively associated. Alternatively, as large numbers of louse eggs should be related to large numbers of blood-feeding nymphs/adults, i.e. those triggering the immune/stimulus itch, a positive relationship between lice load and rates of scratching might indicate that monkeys scratch because of their itch. We then tested a *social hypothesis* that SDB are best explained by social variables alone. This hypothesis is generally related to predicted levels of anxiety and included the following variables: aggression received, social grooming, proximity of higher-ranking individuals, number of neighbours, dominance rank and reproductive status. According to this hypothesis, high-ranking females should be less anxious about social outcomes and interactions because they receive less aggression and have more social options than low-ranking females. If SDB rates are indicators of anxiety, then they should be positively associated with dominance rank, rates of aggression received and proximity to higher-ranking individuals or even neighbours in general. SDB rates should also be higher when females are reproductively active, i.e. when cycling, pregnant or lactating, because they experience changes in their energetic needs, physiology, social interactions, especially increased aggression and coercion from males, and social networks [36–39], changes which can be sources of anxiety and thus be related to changes in SDB rates (e.g. [40]). We also tested an *environmental hypothesis* that SDB are best explained by environmental variables alone, such as seasonality, temperature and rainfall. Ambient temperature and humidity have differential effects on the pelage of animals and on their activities. For instance, according to the environmental hypothesis, we might predict that in summer (short hair), less pelage care is required than in winter (long hair) to achieve the same thermoregulation efficiency. However, hot and humid weather during summer may induce sweating, which may in fact increase the need for pelage care compared with winter. It was thus difficult to predict the sign of the relationship between the occurrence of SDB and environmental factors, so we left predictions open. Furthermore, Japanese macaques are strict seasonal breeders [34], so physiological and behavioural changes are tightly linked to season. In addition, lice loads themselves were shown to vary seasonally in Kōjima macaques [33], so it would be difficult in any case to separate the influence of these factors on SDB.

Because these hypotheses are not mutually exclusive, we also examined the explanatory power of models that included combinations of these main hypotheses. The *parasite–social hypothesis* predicted that a combination of parasitological and social factors best explains scratching and self-grooming. The *parasite–environmental hypothesis* predicted that a combination of parasitological and environmental factors best explains scratching and self-grooming. The *environment–social hypothesis* predicted that a combination of environment and social factors best explains scratching and self-grooming. Finally, the *integrated hypothesis* predicted that SDB are best explained by a combination of parasitological, social and environmental factors. After testing these hypotheses via model comparison, we present the results of the model or set of models that best explained the occurrence of SDB in our observed data.

## 2. Material and methods

### 2.1. Study site, study subjects and data collection

We studied Japanese macaques on Kōjima, a 0.3 km^2^ islet in southern Japan (31°27′ N, 131°22′ E) [41]. Provisioning and behavioural observations of Kōjima macaques started in 1952, and demographic, ecological, behavioural and life-history data are available since then [42]. The study group is currently provisioned with approximately 3 kg of wheat approximately twice weekly.

Data were collected on the 19–20 adult females (more than 7 years old; one female reached adulthood at the beginning of the study and was followed from April onwards) of the main group (approx. 60 individuals in total, including 9 adult males and 23–31 non-adult individuals) from January to November 2014. We focused on females because in Kōjima they form the stable core of the group and dominate dynamics of social networks, whereas males migrate between groups, are often few, peripheral and not very social, and juveniles are difficult to recognize and observe, and usually engage in different age-typical activities than adults (note that intraspecific variation exists in Japanese macaques' social structure [34,42,43]). Observations comprised 1265 15-min focal observations or a mean of 66 (±5 s.d.) per female. Females were observed following a randomized list updated day after day and focal observations were balanced across females and time of day (morning/afternoon). The main activities of females were recorded every minute, while their neighbours in proximity (including within 1, 5 and 10 m) were recorded every 2 min. Females were recorded as reproductively active in the mating (winter) and birth (summer) seasons, according to the occurrence of proceptive behaviours (e.g. approaching and presenting the hindquarters to males), male interest, and copulations, and to the birth and subsequent nursing of an infant respectively, and in the inter-season (spring and autumn) retrospectively if they had given birth. Data on agonistic interactions, i.e. those including bites, chases, hits, threats and displacements/supplantations, were collected during focal observations and ad libitum, and a winner and a loser was determined based on the receiver of the aggressive behaviour fleeing or submitting to her aggressor in order to establish a dominance hierarchy (see below)*.* The number of scratching events and louse egg-picking gestures were counted in the interval between minute-scans. The occurrence of self-grooming was recorded as an activity state (i.e. on the minute-scan), but bouts falling within the interval between minute-scans were also counted in an extra column.

Scratching was operationally defined as moving the fingertips quickly and repeatedly across the same skin area [10,44]. New events started with changes in body area or breaks of more than 5 s (e.g. [25]). A self-grooming bout was defined as an individual grooming herself continuously until she stopped for more than 5 s. Counts of self-grooming bouts falling directly on as well as between minute-scans were pooled for analyses. To estimate lice load, we counted the number of times the groomer conspicuously picked something from the groomee's hair, or her own, for each minute-scan during social and self-grooming bouts. This louse egg-picking behaviour is defined as the groomer focusing on a narrow patch of hair, pinching the base of the hair with the thumb and index fingers or her teeth, pulling the selected object along the length of the hair, and eating the extracted item [30]. This louse egg-picking behaviour is a good estimate of lice load as it has been shown that in 98.9% of the cases, a louse egg is actually picked [30]. Louse egg counts during social grooming were assigned to the female from which eggs were removed, regardless of whether or not she was the focal female (e.g. [33]).

### 2.2. Data analyses

We built our dataset based on our focal observations, which we used as the unit of analysis. For each observation, we computed the variables listed in table 1. Because high numbers of zeros in count variables can lead to modelling issues (e.g. zero-inflation), we transformed several count variables into binary variables, i.e. presence/absence data, coding the occurrence of each behaviour or pattern during the focal observation as 1 and its non-occurrence as 0 (table 1).

**Table 1.** Summary of variables taken into account and their calculations (also see the text). *Per observation* indicates under which form the variable was entered in the models.

scratching	count of scratching events during minute-scans
	per observation: whether (1) or not (0) scratching occurred
self-grooming	sum of counts of self-grooming bouts between minute-scans and of self-grooming bouts falling on a minute-scan and written as an activity
	per observation: whether (1) or not (0) self-grooming occurred
lice load	count of louse egg-picking gestures during grooming divided by the number of grooming minute-scans
	per observation: monthly average
social grooming	minute-scan record of whether the focal individual grooms or is groomed by another individual
	per observation: whether (1) or not (0) social grooming occurred
aggression received	the focal individual receives a threat, a chase, a hit or a bite from another individual during either its focal observation or ad libitum
	per observation: whether (1) or not (0) the focal individual received aggression, separately during focal and ad libitum
provisioning day	the main group is regularly provisioned with 3 kg of wheat grains. Wheat is thrown on the sand of the main beach of the island over a limited area, which creates an increased potential for aggression to occur compared with when provisioning does not occur
	per observation: whether (1) or not (0) the group was provisioned on that day
dominance rank	dominance rank as determined by the calculation of David's scores (see the text)
	per observation: David's score of the focal individual (number between 1 and *N* − 1)
proportion of higher-ranking females within 10 m proximity	number of proximity scans with females that are higher ranking than the focal female as a proportion of all proximity scans with females as neighbours
	per observation: proportions between 0 and 1
number of female neighbours within 5 m proximity	number of different females within 5 m proximity for each proximity scan
	per observation: sum of those numbers (number between 0 and maximum 152 (19 potential female neighbours times 8 proximity scans))
reproductive status	reproduction is seasonal in Japanese macaques but females do not cycle every year and although they did cycle during the mating season, they may not become pregnant and give birth. Their reproductive status can thus vary
	per observation: whether (1) or not (0) the focal female was reproductively active, i.e. either cycling, pregnant or lactating
rainfall	total amount of rain in millimetres per day over the entire study period
	per observation: average amount of rain in millimetres over 3 days including 2 days before and the day of observation
temperature	average temperature in °C per day over the entire study period
	per observation: average temperature of the day of observation
season	climatic season during which the observations were carried out
	per observation: winter, spring, summer, autumn

#### 2.2.1. Parasitological variables

Models including these variables, alone or in combination with others (table 1), tested the parasitic hypothesis that the occurrence of scratching and self-grooming is related to lice load.

The frequency of louse egg-picking gestures by unit of grooming received served as a proxy for louse infestation [33]. Females collected an average of 0.77 louse eggs per grooming minute-scan (median, range = 0.3–2.23, *N* = 20, 1885 louse egg-picking gestures in 5647 grooming minute-scans and 397 in 975 self-grooming minute-scans) [33]. Lice load was calculated as monthly average values of louse egg-picking counts divided by number of grooming minute-scans [33]. A month was the shortest timeframe under which lice load was accurately determined (i.e. the average per individual did not change after between 7 and 11 days of observation, and 11 days of observation sometimes constituted a whole month of data collection due to inconsistent access to the island).

#### 2.2.2. Social variables

Models including these variables, alone or in combination with others (table 1), tested the social hypothesis that scratching and self-grooming are related to social factors and representative of social uncertainty or anxiety.

Social grooming reduces anxiety because it is linked with the release of rewarding opioid neuropeptide beta-endorphins [45,46] and has been connected with a reduction in heart rate [14,47] and SDB [10,16,22]. We thus included the occurrence of social grooming in the models as it is possible that it influences the likelihood of occurrence of SDB compared to observations where social grooming did not occur.

Dominance rank is associated with social uncertainty because low-ranking individuals are more likely to receive aggression (in this study, correlation between rank and aggression received: *r*_Pearson_ = −0.51, *t* = −2.54, d.f.= 18, *p* = 0.020, *N* = 20). In a socially strict system such as that of Japanese macaques, low-ranking individuals are more constrained in their behavioural options than high-ranking individuals are [48]. Dominance rank was assigned through the calculation of normalized David's scores (normDS), an individual score of relative power based on the successes (winning versus losing) of an individual in agonistic interactions while accounting for the other group members' successes [49]. Calculations were based on matrices of decided agonistic interactions. The highest-ranking female receives the highest score.

To calculate individual rates of aggression received (number of events divided by observation time), we only considered focal data. This variable was then transformed into a binary variable, with the focal female either receiving or not receiving aggression. From the ad libitum data of each observation day, we additionally coded whether or not the focal female received or did not receive aggression during that day of observation, notably in order to account for the increased likelihood of aggression occurrence on provisioning days. The occurrence of provisioning on each observation day was also therefore included as a control factor. At the study site, provisioning involves providing the group with a small amount of wheat over a short duration in a limited area, which dramatically increases the frequency of aggression for the majority of the group and may thus have an influence on behaviours sensitive to social conditions.

The presence of high-ranking individuals has been shown to be a factor in social uncertainty inasmuch as their proximity can increase the rates of SDB [7,50,51]. We calculated the number of proximity scans up to 10 m in which higher-ranking females were present as a proportion of all scans in an observation, thereby giving per observation a number between 0 and 1. We also counted the number of different female neighbours within 5 m proximity for all proximity scans in an observation. We chose two different proximity thresholds, a radius of 5 m proximity representative of social integration and a radius of 10 m representative of social uncertainty potential. This was based on the facts that first, Japanese macaques living under natural conditions seem to tolerate each other without aggression above a proximity threshold of 1 m [52]. Second, given the high proportion (20%) of negative social interactions resulting from entering the proximity of another individual [48], it is fair to assume that the approach of a higher-ranking individual as far as 10 m can already potentially create uncertainty as to how this animal will behave.

We finally included the reproductive status of the females as either active, i.e. cycling, pregnant or lactating, or inactive. Indeed, reproductive activity is seasonal (with winter and summer being the mating and birth season respectively, with variation throughout Japan [34]) and induces drastic changes in the females' behaviour and physiology [36–38,53], which may influence rates of SDB, either directly or through interactions between reproductive state and social interactions, seasonal factors and/or lice load.

#### 2.2.3. Environmental variables

Models including these variables, alone or in combination with others (table 1), tested the environmental hypothesis that scratching and self-grooming are related to climatic factors.

Daily rainfall and daily average temperatures were extracted *a posteriori* from the historical records of a meteorological service online provider (http://www.accuweather.com/en/jp/aburatsu/219041/weather-forecast/219041) based on data from the weather station nearest to the field site and on the same side of the coast (Aburatsu, 25 km). Because access to the island for observation was limited to days with relatively good weather (i.e. little rain or strong winds), thereby introducing a bias towards having no rain, we used the mean rainfall over three days including the two days preceding the observation and the day of observation itself. We also included the categorical variable season (winter, spring, summer, autumn) as Japanese macaques are highly seasonal animals at many levels (reproduction, moulting, sociality, etc.) [34].

The dataset is provided in electronic supplementary material, table S1.

### 2.3. Statistical analyses

Analyses were carried out in R v. 3.1.2 [54]. We ran generalized linear mixed models with a binomial error structure and logit link function with the function glmer from the lme4 package [55]. Models are presented in table 2. Focal animal identity, date and time of day (morning/afternoon) were included as random factors to control for pseudo-replication and the effect of time of day on social interaction and the frequency/occurrence of SDB [7,9,50,56]. Model assumptions (homogeneity of residuals, variance inflation factors below or around 1, and stability of estimates [57]) were tested and found to be fulfilled and no influential cases were detected.

**Table 2.** List of models included in the comparison.

	independent variables											
testing the hypothesis(ses)	lice load	social grooming	aggression received (focal)	aggression received (ad libitum)	feeding day	David's score	prop. higher-rank nn10	number females nn5	reproductive status	rainfall	temperature	season
integrated	x	x	x	x	x	x	x	x	x	x	x	x
parasitic	x											
social		x	x	x	x	x	x	x	x			
environmental										x	x	x
parasitic--social	x	x	x	x	x	x	x	x	x			
parasitic--environmental	x									x	x	x
environmental–social		x	x	x	x	x	x	x	x	x	x	x

To compare all alternative hypotheses simultaneously and objectively, we used an information-theory approach based on Akaike's information criterion (IT-AIC) which provides an objective ranking of models from a candidate set and an estimation of their relative explanatory values [58]. The principle of this approach relies on assessing the likelihood and uncertainty of one or several models in a candidate set to represent the ‘reality’ or ‘truth’. This can be judged by the AIC value as well as the difference in AICs between the model with the smallest AIC and the others (AICs in increasing order) and the likelihood and evidence ratio of each model compared with the one with the smallest AIC value [58]. In this way, we obtain a formal strength of evidence for each candidate model linked to a specific hypothesis. With the package AICcmodavg (function aictab) [59], we extracted the AIC of each model and ranked them accordingly. Convention sets a difference in AIC of more than two as indicative of a model having stronger explanatory value than another; we nevertheless considered models with AIC differences of up to four points as parsimonious candidate models to be conservative [58]. The function aictab also computes each model's Akaike's weight, or relative likelihood, which indicates to what extent one model is more likely than another in the candidate set to provide a reasonable explanation of the variance in the data. Akaike's weights were then used to compute evidence ratios (equal to the weight of the model with the lowest AIC divided by the weight of the model to compare it against), which determine the extent to which one model had stronger explanatory value over another, if any. We then used the modavg function of the same package to extract weighted parameter estimates, unconditional standard errors and 95% CIs of all predictor variables repeatedly occurring within the set of candidate models. Parameter estimates can be averaged across all models in the candidate set (full averaging), even those in which the variable of interest does not appear (in which case parameters are set to zero) or only across models in which the variable of interest appears (conditional averaging) [58]. We chose the latter strategy because we had strong *a priori* reasons to include specific variables in specific models. We also chose to show average parameter estimates instead of only those parameters estimated from the model with lowest AIC because, although the different models offer different interpretations of the data, all interpretations from models within the candidate set are plausible.

## 3. Results

Female Japanese macaques of Kōjima scratched on average 6.9 times per hour of observation (median, range = 3.7–11.0, *N* = 20) and groomed themselves 4.5 times per hour of observation (median, range = 2.0–5.7, *N* = 20).

**Figure 1.**
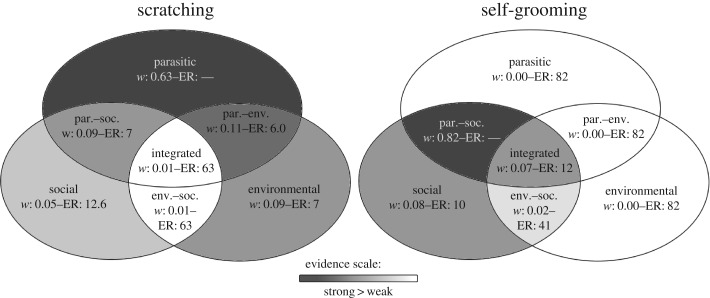
Schematic presentation of results. With the name of the model are given Akaike's weight (*w*) and evidence ratio (ER) (null ‘—’ for the ‘best’ model) of each model. Colour darkness indicates level of support, from dark grey (model with the highest *w*) to white (model with lowest *w*) with intermediate *w* and ER in shades of grey in descending order of importance.

Among the candidate models with the occurrence of scratching as the response variable, the parasite model with monthly lice load had the lowest AIC value and a weight of 0.63, followed by models including parasitological and social or environmental variables as well as environmental variables only (cumulative Akaike's weight of 0.92; ΔAIC up to 3.97; table 3 and figure 1). Within the model candidate set, the parasite model had 5.7–7.0 times more empirical support than the three closest competing models, i.e. those with the next lowest AICs (table 3 and figure 1). In models including lice load as a predictor, females were more likely to scratch if they had higher monthly lice loads (averaged *β* = 0.26 ± 0.15 unconditional s.e., unconditional 95% CI = 0.02–0.55; table 4). Among social factors, there was a small tendency for the number of neighbours within 5 m proximity to increase the occurrence of scratching (averaged *β* = 0.02± 0.01 unconditional s.e., unconditional 95% CI = −0.01–0.04; table 4). The occurrence of scratching was also positively associated with the occurrence of social grooming (averaged *β* = 0.29± 0.14 unconditional s.e., unconditional 95% CI = 0.01–0.56; table 4). Other social variables and environmental factors explained little to none of the variance in the data (table 4).

**Table 3.** Model characteristics. *K*, number of variables included; AIC, Akaike's information criterion; ΔAIC, difference in AIC between the model with the lowest AIC and the target model; weight, model probabilities (*sensu* Burnham & Anderson [58]); cum. weight, cumulative weight; ER, evidence ratio: weight of the model with the lowest AIC divided by weight of the target model. Models in italics are those within ΔAIC < 4 of the model with the lowest AIC (see the text and Burnham & Anderson [58]). Abbreviations: par, parasitic; soc, social; env, environmental.

models	*K*	AIC	ΔAIC	weight	cum. weight	log-likelihood	ER
*scratching*							
parasitic	*5*	*1499*.*94*	*0*	*0*.*63*	*0*.*63*	−*744*.*97*	*—*
par–env	*10*	*1503*.*44*	*3*.*51*	*0*.*11*	*0*.*74*	−*741*.*72*	*5*.*7*
par–soc	*13*	*1503*.*88*	*3*.*95*	*0*.*09*	*0*.*83*	−*738*.*94*	*7*
environmental	*9*	*1503*.*91*	*3*.*97*	*0*.*09*	*0*.*92*	−*742*.*95*	*7*
social	12	1504.84	4.90	0.05	0.97	−740.42	12.6
env–soc	17	1507.74	7.80	0.01	0.99	−736.87	63
integrated	18	1507.79	7.86	0.01	1.00	−735.90	63
*self-grooming*							
par– soc	*13*	*1492*.*37*	*0*	*0*.*82*	*0*.*82*	−*733*.*19*	*—*
social	12	1497.08	4.71	0.08	0.90	−736.54	10.3
integrated	18	1497.18	4.81	0.07	0.98	−730.59	11.7
env– soc	17	1499.50	7.13	0.02	1.00	−732.75	41
parasitic	5	1565.40	73.03	0.00	1.00	−777.70	>82
par– env	10	1570.78	78.41	0.00	1.00	−775.39	>82
environmental	9	1570.94	78.57	0.00	1.00	−776.47	>82

**Table 4.** Multi-model inference results: model averaged parameter estimates (*β*) ± unconditional standard errors (s.e.) (95% unconditional confidence intervals CI). In italics are variables for which CI does not include zero. Variable parameters are averaged only over models in which the variable appears, except for the intercept's, averaged across all models (see the text).

	scratching *β* ± s.e. (95% CI)	self-grooming *β* ± s.e. (95% CI)
intercept	0.51 ± 0.31 (−0.10–1.12)	−0.53 ± 0.49 (−1.48–0.43)
monthly lice load	*0.26 *±* 0.15 (0.02*–*0.55*)	−*0.27 *±* 0.14* *(−0.53–0.00)*
social grooming	*0.29 ± 0.14 (0.01–0.56)*	*1.00* ± *0.14* (*0.74*–*1.27*)
aggression received (focal)	0.14 ± 0.20 (−0.25–0.53)	0.32 ± 0.19 (−0.05–0.70)
aggression received (ad libitum)	0.12 ± 0.15 (−0.19–0.42)	0.13 ± 0.15 (−0.16–0.43)
feeding day	0.18 ± 0.17 (−0.16–0.52)	0.19 ± 0.16 (−0.11–0.50)
David's score	−0.01 ± 0.02 (−0.05–0.02)	−*0.03 ± 0.01 (−0.05–0.00)*
prop. high-rank nn10	0.14 ± 0.16 (−0.18–0.46)	−0.20 ± 0.16 (−0.52–0.11)
nb females nn5	0.02 ± 0.01 (−0.01–0.04)	*0.05 ± 0.01 (0.02–0.07)*
reproductive status	−0.09 ± 0.18 (−0.45–0.27)	−0.01 ± 0.14 (0.28–0.27)
rainfall (3 days)	0.00 ± 0.01 (−0.02–0.01)	0.00 ± 0.01 (−0.01–0.02)
temperature	0.03 ± 0.02 (−0.02–0.07)	0.03 ± 0.02 (−0.02–0.07)
season winter	−0.37 ± 0.34 (−1.03–0.30)	0.58 ± 0.32 (−0.05–1.21)
season spring	0.00 ± 0.21 (−0.40–0.41)	0.28 ± 0.19 (−0.09–0.66)
season summer	−0.15 ± 0.32 (−0.78–0.47)	−0.23 ± 0.29 (−0.80–0.34)

Among the candidate models with the occurrence of self-grooming as the response variable, the parasite–social model with parasitological and social variables had the lowest AIC value and an Akaike's weight of 0.82. Within the candidate set, the parasite–social model had 10.3 times more empirical support than the second model in the list (table 3 and figure 1). Within this model, the occurrence of self-grooming was negatively associated with monthly lice load (av. *β* = −0.27± 0.14 unc. s.e., unc. 95% CI = −0.53–0.00; table 4). Females were more likely to groom themselves if social grooming occurred (av. *β* = 1.00± 0.14 unc. s.e., unc. 95% CI = 0.74–1.27; table 4), if they had a higher number of female neighbours within 5 m proximity (av. *β* = 0.05 ± 0.01 unc. s.e., unc. 95% CI = 0.02–0.07; table 4) and if they were low ranking (av. *β* = −0.03 ± 0.01 unc. s.e., unc. 95% CI = −0.05–0.00; table 4).

## 4. Discussion

SDB such as scratching and self-grooming can be explained by a number of factors related to parasites, sociality and the environment. Often enough, studies focus on a single hypothesis only. Taking an integrative approach and examining all hypotheses simultaneously and objectively, this study shows that in female Japanese macaques at Kōjima, scratching and self-grooming occurrences are better explained by models including lice load and social factors than other combinations of variables.

Within models including lice load, the occurrence of scratching was positively associated with monthly lice loads. Chemicals in saliva, stings, body secretions or urticating hairs of ectoparasites all have the potential to induce an immune itch which triggers scratching, effectively relieving the itch [1,17]. Additionally, although scratching may not remove the egg from its position on the hair or feather, it may damage it and halt its development [5], adding a prophylactic benefit similar to that of self-grooming with the extra advantage that with scratching, an animal can reach areas inaccessible to self-grooming [5]. This link is commonly established in many animals such as ungulates and birds [3–5] but is neglected in primates because, among other reasons, they are social animals and scratching was linked early on to social events and anxiety due to social events.

Inversely, the occurrence of self-grooming was negatively linked to monthly lice loads. By grooming themselves, females thus may be able to prevent infestation by removing future blood-sucking ectoparasites [4]. However, the occurrence of self-grooming was also linked to the occurrence of social grooming, larger numbers of female neighbours in relatively close proximity, as well as to lower dominance rank. These results therefore also support the hypothesis that, in addition to its original prophylactic function, self-grooming may act as a displacement activity that could potentially provide an escape from socially uncertain situations [11]. For instance, Japanese macaque social behaviour is highly biased towards kin so that individuals found often in proximity of each other are likely to be genetically related to a high degree [48]. Given that matrilines are rather small (between two and four adult females) and few (three) in the study group, larger numbers of female neighbours could be linked to the increased presence of non-kin in proximity which could be related to social uncertainty and bouts of self-grooming. Future studies could investigate the effect of the presence of kin versus non-kin in relation to SDB when possible.

A major factor positively associated with the occurrence of SDB was the occurrence of social grooming. Several studies have actually reported a decrease in SDB with the occurrence of grooming in accordance with its proposed role in tension reduction [10,16,22]. However, the occurrence of social grooming may intensify the expression of SDB, a pattern that is hypothesized to relate to the risk of aggression due to increased proximity (e.g. [60]), the uncertainty at the beginning or end of a grooming bout in terms of activity change or social situation (e.g. [10,23]), or behavioural transitions that could be facilitated by SDB (as displacement activities) (e.g. [61]). Other hypotheses for increased SDB in this context that have rarely been considered include the fact that animals may experience some kind of behavioural contagion, simply copying the activities of others or wanting to prolong grooming (e.g. [44]), or they may still feel the touch of the grooming activity on their skin or a disturbance in hair arrangement (e.g. [61]). All these explanations remain speculative pending further investigation, but it is noteworthy that the relevant stimulus, a mild mechanical touch and/or a change of temperature (due to body contact or disturbance of the hair or feathers), has the potential to activate the same neural sensory afferent fibres (C fibres), i.e. those involved in the sensation of pain, temperature, touch and itch [62,63].

Interestingly, social and environmental factors that we investigated had less weight in explaining variation in SDB when compared to lice load. This is despite the fact that Japanese macaque society is governed by strict rules following dominance and kinship relationships where individuals are constrained in their behavioural options [48,64] and tightly linked to a seasonally changing environment impacting reproduction and sociality [34]. Although previous studies on primates have linked increased urinary cortisol levels (an indicator of unbalanced homeostasis or stress) and increased scratching rates to active reproductive state [39,40], and increased rates of SDB to challenging weather [9], those variables accounted for little to none of the variation in SDB in female macaques of Kōjima. One explanation could be that the measured variables are too coarse (either reproductively active or inactive over the season and average rain amount over 3 days) to detect any meaningful pattern. Concerning the apparent lack of effect of reproductive status on SDB, another explanation could be linked to the seasonality of reproduction. During the mating season, many females are cycling at the same time and many males get an opportunity to mate; as such, the degree of competition for reproduction can be considered moderate [65]. Thus, although females are more active than when they are not reproductively active, they may have means or opportunities to avoid stressful situations like male coercion, for example by isolating themselves from the group to copulate with a male of their choice [66].

It could thus be the case that animals scratch primarily because of an immune/stimulus itch triggered by ectoparasite bites/movements. Nevertheless, this primary explanation is not exclusive of the fact that animals can scratch because of an idiopathic non-immune itch, e.g. if they are anxious in a given situation or if the atmosphere is hot and humid. The endocrine system is implicated in the regulation of internal states and behaviours [67] and is linked to the immune system [68]. Long-term release of ‘stress’ hormones (glucocorticoids), whether linked to social or environmental factors, tempers immune function and decreases its efficacy, probably making animals more susceptible to infections from diverse parasites/pathogens [68]. Thus, an anxious animal or an animal in a challenging environment could also be a lousier animal because of a generally weakened state.

The prophylaxis/parasitic hypothesis can actually embody altogether several reasonable explanations for variation in SDB inasmuch as ectoparasites are often transferred from one host to the next through body contact between hosts [1,18]; they greatly depend—sometimes solely (e.g. louse)—on their hosts for reproduction and survival [1,18]; they are susceptible to seasonal changes due either to their own biology, that of the host or that of the environment [28,69]; and through their blood meal they may be sensitive to the physiological state of their hosts [19,70], which may in turn be dependent on environmental and social conditions [36–38,71]. Revisiting studies linking SDB changes to environmental or social changes taking into account ectoparasite loads could fill the gaps in our knowledge of mechanisms or functions that we are still unable to explain fully, for example considering the inconsistent results about the links between social grooming and scratching, or the so-far under-investigated difference between a stimulus and an idiopathic itch, or the inclusion of a broader range of ectoparasites such as ticks and fleas (e.g. [8,27,72,73]).

Previous research often examined each of the tested hypotheses separately. Our results attest to the fact that studies should not discount the importance of hygienic/prophylactic functions of behaviour, even when testing ideas linked to social processes. It is indeed more likely that a diversity of factors affects the behaviour of animals, sometimes synergistically, sometimes independently. Taking an integrative approach thus allows for a holistic view of animal behaviour. This is facilitated by the information-theory framework used in this paper and advocated by Burnham & Anderson and others [58,74–76]. In doing so, deeper integrative insights into an animal's biology are attained, which provides a basis for further investigation. Furthermore, the investigation and use of non-invasive indicators of ectoparasite infestation, like that used in this study, can bring about further understanding of wildlife epidemiology, infection risk and links between sociality and health.

## Supplementary Material

ESM TableS1 dataset to the article Scratch that itch Duboscq et al

## Data accessibility

Data are provided in the electronic supplementary material.
